# A giant chylolymphatic cyst of the retroperitoneum: a case report

**DOI:** 10.1093/jscr/rjad320

**Published:** 2023-06-07

**Authors:** Bana Zuhair Alafandi, Marwan Al Aliwy, Raghad Hakim, Laura Merjaneh, Mohamed Muhanad Balid, Joud Markaby, Linda Shehade, Sarab Agha, Kusay Ayoub

**Affiliations:** Faculty of Medicine, Aleppo University, Aleppo, Syria; Department of General Surgery, Aleppo University Hospital, Aleppo, Syria; Faculty of Medicine, Aleppo University, Aleppo, Syria; Department of Gastroenterology, Alrazi Hospital, Aleppo, Syria; Faculty of Medicine, Aleppo University, Aleppo, Syria; Faculty of Medicine, Aleppo University, Aleppo, Syria; Department of Pathology, Aleppo University Hospital, Aleppo, Syria; Department of Pathology, Aleppo University Hospital, Aleppo, Syria; Department of General Surgery, Aleppo University Hospital, Aleppo, Syria

**Keywords:** chylolymphatic cyst, retroperitoneum, intermittent claudication, traumatic, recurrence, mesenteric cyst

## Abstract

Chylolymphatic cysts are an extremely rare variant of mesenteric cysts and account for 7.3% of all abdominal cysts. They can develop anywhere along the mesentery of the gastrointestinal tract and present in a wide range of symptoms. A 46-year-old male presented with mild abdominal pain and intermittent claudication in his right leg for the last 2 months and a history of a retroperitoneal resection of a simple abdominal cyst 5 years ago. Abdominal ultrasound and computerized tomography showed a fluid-filled cystic lesion measuring 17 × 11 × 10 cm in the right retroperitoneum. The cyst was surgically excised, and the histopathological examination was consistent with the chylolymphatic cyst. On a 1-year follow-up, the patient is recovered with no recurrence observed. Our report presents a case of a giant retroperitoneal chylolymphatic cyst with uncommon presenting symptoms and a rare etiology.

## INTRODUCTION

Mesenteric cysts are rare intra-abdominal lesions with an incidence rate of 1:100 000 in adults and 1:20 000 in pediatric admissions [1]. Chylolymphatic cysts are a rare variant of mesenteric cysts and were first reported in 1842 by Rokitansky [2]. They are more common in the pediatric population and can develop anywhere along the mesentery of the gastrointestinal tract. Their pathology remains unclear, and the symptoms vary according to the size and location of the cyst. The diagnosis is confirmed on microscopic examination, and the treatment of choice is complete surgical excision with regular follow-up imaging despite the low rates of recurrence. We, herein, present a case of a giant retroperitoneal chylolymphatic cyst in a 46-year-old male presenting in uncommon compressive symptoms.

## CASE PRESENTATION

**Figure 1:**
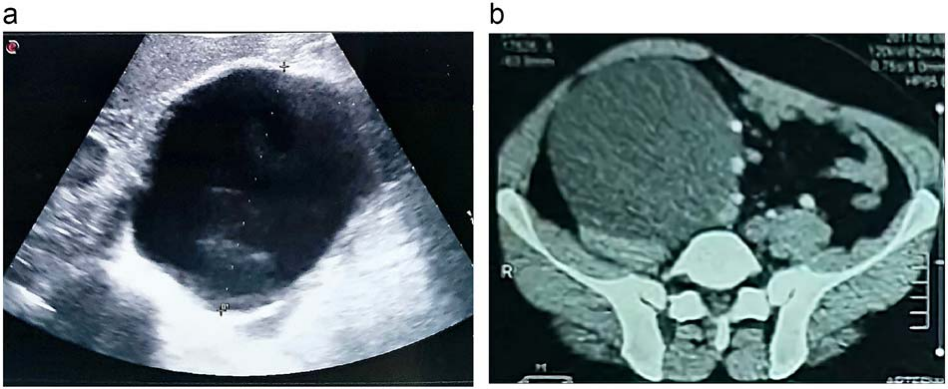
**a.** An US image showing a large cystic mass in the right abdomen. **b.** A CT scan of the abdomen showing a cystic mass of 17 × 11 × 10 cm with a fat-fluid level.

**Figure 2:**
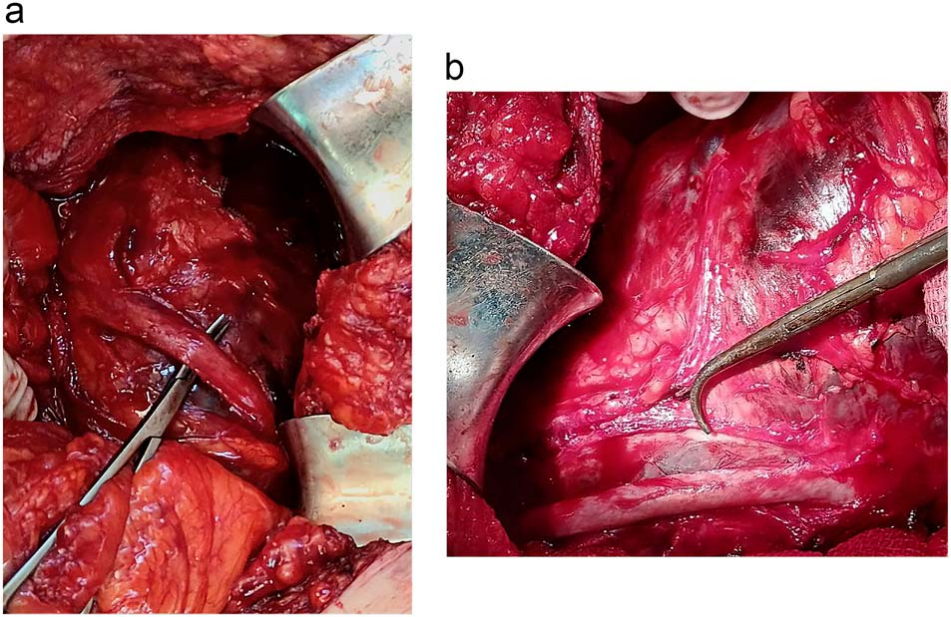
**a**. Isolation of the right ureter. **b.** Isolation of the right iliac artery.

**Figure 3:**
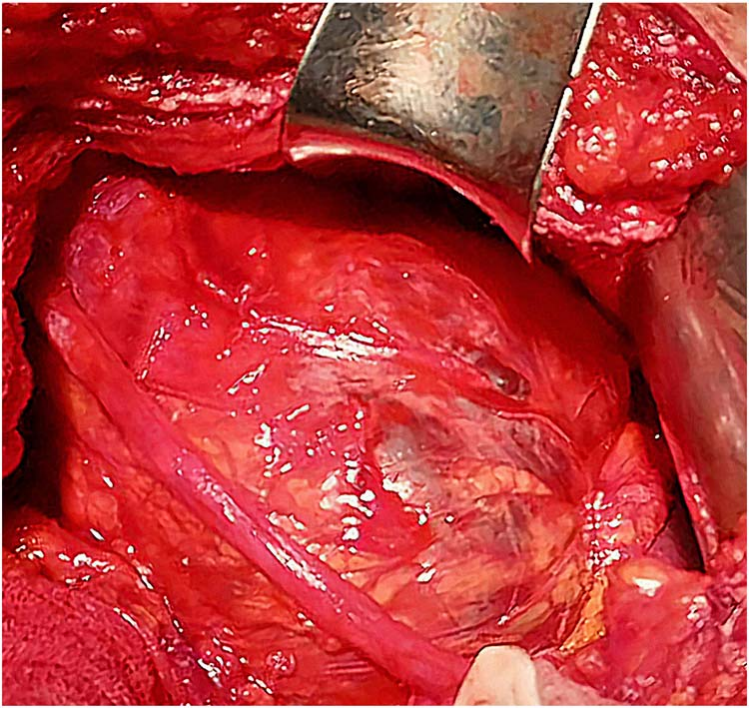
Intraoperative view of the cyst.

**Figure 4:**
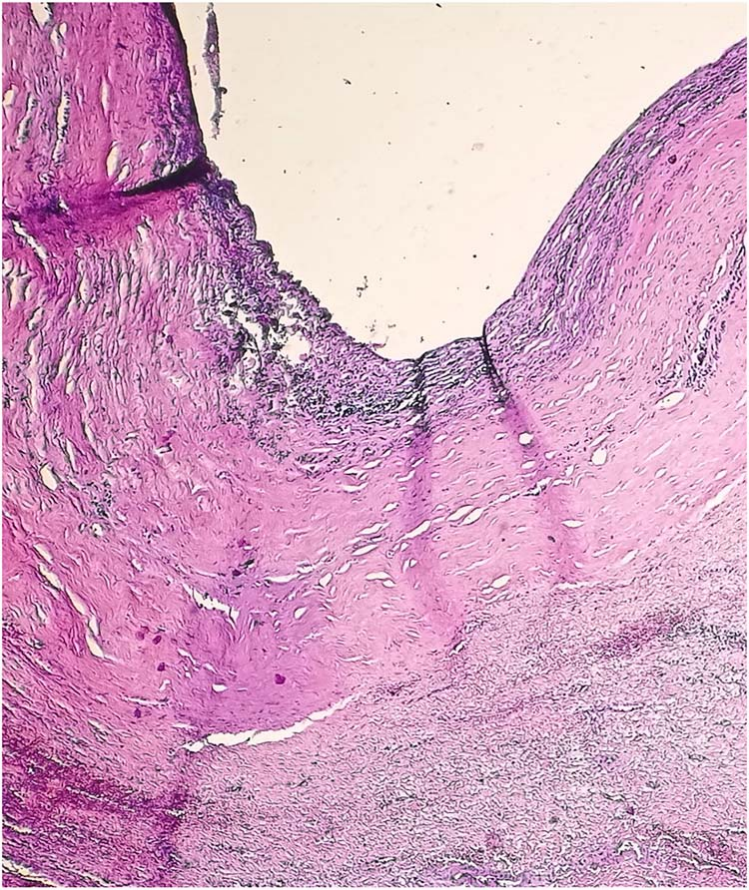
Microscopic view of the chylolymphatic cyst wall.

A 46-year-old male presented with mild abdominal pain for the last 2 months. The pain was generalized, recurrent and did not relieve on medication. The patient showed intermittent claudication in his right leg. He presented with a similar complaint five years ago; abdominal pain and urinary urgency and hesitancy, which led to a diagnosis of a simple abdominal cyst. Past medical history was insignificant. Surgical history included retroperitoneal cystectomy, herniorrhaphy and a cleft palate repair. Patient had no other complaints and was not on any medications. He was a smoker with a 22 pack-year smoking history. His temperature was normal, and there was no history of weight loss or loss of appetite or change in bowel movement. On physical examination, the abdomen was soft and non-tender without distention. There was a palpable mass in his right flank. Vital signs and blood tests were within normal limits. Abdominal ultrasound (US) showed a large fluid-filled cystic lesion (Fig. 1a). Subsequent computerized tomography (CT) of the abdomen showed a large, well-defined, clear fluid-filled cyst measuring 17 × 11 × 10 cm, located in the retroperitoneum abutting the lower half of the right kidney, extending to the pelvis and compressing the inferior vena cava (Fig. 1b). Patient was cleared for surgery. Surgical exploration showed several adhesions surrounding the cyst. The cyst was partially isolated from the right ureter and iliac vein and artery (Fig. 2a and b), then surgical excision of 90% of the cyst was performed (Fig. 3). Histopathological report found the cystic wall composed of fibrous connective tissue and lined by one layer of flat cells, with mild lymphocytic infiltrate and congested blood vessels confirming the diagnosis of a chylolymphatic cyst (Fig. 4). On a 1-year follow-up, the patient appears to be recovered and in good health with no recurrence.

## DISCUSSION

Chylolymphatic cysts are an extremely rare variant of mesenteric cysts, and account for 7.3% of all abdominal cysts. They might develop anywhere within the mesentery of the gastrointestinal tract. Chylolymphatic cysts have variable clinical manifestations, ranging from common to rare presentations, depending on the cyst’s size, location and age at presentation. They commonly present with abdominal pain or abdominal distention, to more severe cases presenting with an acute abdomen secondary to intestinal obstruction, hemorrhage or rupture [3]. Mesenteric cysts are usually symptomatic in children, and commonly present in acute abdomen [4], whereas in adults, cysts are found incidentally in 40% of cases, as they tend to mimic other pathologies and are often asymptomatic while they grow in size [5].

Our case highlights uncommon symptoms. The presentation of intermittent claudication in a 46-year-old male was indicative of an abdominal mass causing compressive effects, which was confirmed by an abdominal US and a CT scan.

Mesenteric cysts are most commonly found in the mesentery of the small intestine (60%), followed by the large intestine (24%) and the retroperitoneum (14.5%) [6]. Although the retroperitoneum is a relatively common location for mesenteric cysts, cases of retroperitoneal chylolymphatic cysts are seldom reported in the literature.

Recurrence of mesenteric cysts is uncommon; however, it was significantly correlated with the location of the cyst, and is more likely to occur in retroperitoneal cysts due to the proximity of major vessels and visceral organs, which prevents complete excision, subsequently increasing recurrence rate [6].

Chylolymphatic cysts of size >15 cm are considered giant, and due to the location of the cyst in the retroperitoneum, our patient developed compressive symptoms that lead to its early detection despite having initially presented with mild abdominal pain, which was not characteristic for any specific disease.

Chylolymphatic cysts were studied in adult and pediatric populations separately, which resulted in different characteristics accordingly. In children, the mean age of presentation is 4.9 years with a slight male predominance [7, 8], whereas in adults, both sexes are affected equally and are often diagnosed in the fifth decade [3, 9]; our patient falls within this range.

Chylolymphatic cysts were classified based on underlying etiologic factors into four groups: congenital and developmental cysts, traumatic or acquired cysts, neoplastic cysts and infective and degenerative cysts [10]. Chylolymphatic cysts of traumatic origin are not common as most reported cases were developmental. Moreover, the development of a traumatic chylolymphatic cyst after surgery due to complications of the lymphatic system is a very rare event; only one study reported a chylolymphatic cyst following a fundoplication [11].

Accordingly, the findings of this case suggest a chylolymphatic cyst of traumatic origin, considering the histopathologic features of the cyst wall consisting of fibrous connective tissue and infiltrated by lymphocytes and congestive blood vessels, and a history of a surgical intervention on the same location resulting in trauma to the lymphatic channels.

Another possible etiology is the recurrence of the previous retroperitoneal cyst.

Complete excision of these cysts is the treatment of choice, however, due to the huge size and location of the cyst in the retroperitoneum, partial excision through an open laparotomy was the proper choice of management. The early diagnosis and management despite subtle presenting symptoms yielded into complete recovery with an uneventful 1-year follow-up.

In conclusion, notwithstanding its rarity, diagnosis of chylolymphatic cysts should be considered whenever an abdominal mass is recognized regardless of the patient’s age or gender. Regular follow-up imaging is recommended considering the risk of recurrence. We accentuate the importance of reporting new emerging cases in unravelling clinical obscurities related to chylolymphatic cysts.

## Data Availability

All data supporting the findings of this study are included within the paper.
